# Untreated Graves’ Disease Complicated by Thyroid Storm and High-Output Cardiac Failure: A Case Report

**DOI:** 10.7759/cureus.58205

**Published:** 2024-04-13

**Authors:** Maitree Patel, Ali Z Ansari, Srihita Patibandla, Kirk Moses

**Affiliations:** 1 Internal Medicine, Merit Health Wesley Hospital, Hattiesburg, USA; 2 Pathology, William Carey University College of Osteopathic Medicine, Hattiesburg, USA; 3 Internal Medicine, Trinity Health Grand Rapids, Grand Rapids, USA; 4 Internal Medicine, Merit Health Wesley, Hattiesburg, USA

**Keywords:** high-output heart failure, abdominal pain, cardiomegaly, pericardial effusion, clostridium difficile, thyrotoxicosis, rapid ventricular response, atrial fibrillation, graves disease, thyroid storm

## Abstract

Thyroid storm is a rare yet critical complication of uncontrolled thyrotoxicosis, posing significant challenges in clinical management. We present the case of a 65-year-old African-American female with a medical history significant for untreated Graves' disease, hypertension, and diverticulosis, who presented with escalating abdominal pain, accompanied by nausea, vomiting, diarrhea, and chest discomfort. Upon admission, she exhibited atrial fibrillation with rapid ventricular response (RVR) and newly diagnosed high-output cardiac failure. Diagnosis of thyroid storm was confirmed through comprehensive laboratory assessments and clinical evaluation. Treatment with beta-blockers, anti-thyroid medications, and corticosteroids facilitated stabilization of her condition. This case report highlights the importance of early identification and intervention in thyroid storm to avert potential morbidity and mortality.

## Introduction

Thyroid disorders encompass a spectrum of conditions, ranging from benign nodules to life-threatening thyroid storms. Among these, thyroid storm represents a rare but critical complication of uncontrolled thyrotoxicosis, with potentially fatal consequences if left untreated [1]. The precise incidence of thyroid storm is not well-documented, but in the general population, it is estimated at 0.57-0.76 per 100,000 people per year in the United States of America (USA) and 0.20 per 100,000 people per year in Japan. In hospitalized patients, the incidence is higher, ranging from 4.8-5.6 per 100,000 per year, and thyroid storm occurs in 1%-2% of those admitted for thyrotoxicosis. The mortality rate for hospitalized patients with thyroid storm is approximately 75%, with an overall mortality rate of 10%-20%. The leading causes of death include multiple system dysfunction, heart failure, respiratory failure, and sepsis [2]. Graves' disease, an autoimmune disorder characterized by the production of stimulating autoantibodies against the thyrotropin receptor, accounts for most cases of thyrotoxicosis [3]. It predominantly affects women, with a peak incidence in the third to sixth decades of life [4]. The dysregulation of the hypothalamic-pituitary-thyroid axis in Graves' disease leads to excessive synthesis and release of the thyroid hormones, triiodothyronine (T_3_) and thyroxine (T_4_), resulting in a hypermetabolic state.

Thyrotoxicosis manifests clinically with symptoms including palpitations, heat intolerance, weight loss, tremors, and proximal muscle weakness [5]. However, thyroid storm represents an extreme manifestation of thyrotoxicosis, precipitated by factors such as infection, surgery, trauma, or discontinuation of antithyroid medications [6]. It is characterized by a sudden and severe exacerbation of symptoms, leading to multi-organ dysfunction and hemodynamic instability. Common clinical features of thyroid storm include tachycardia, fever, gastrointestinal disturbances (e.g., nausea, vomiting, diarrhea), altered mental status, and cardiovascular collapse [7]. Notably, patients with Graves' disease are particularly susceptible to thyroid storm due to the underlying autoimmune process and the potential for abrupt changes in thyroid hormone levels [8]. Here, we report a case of thyroid storm in a patient with untreated Graves' disease presenting with abdominal symptoms, atrial fibrillation with rapid ventricular response (RVR), and new-onset high-output cardiac failure.

## Case presentation

A 65-year-old African-American female, known to have untreated Graves' disease, hypertension, and diverticulosis, presented to the emergency department (ED) with escalating abdominal pain, accompanied by nausea, vomiting, diarrhea, and chest discomfort. On admission, her vital signs were notable for a blood pressure of 153/103 mmHg, heart rate of 163 beats per minute (bpm), and oxygen saturation of 100% on room air. Initial laboratory investigations revealed a hemoglobin level of 9.4 g/dL and a magnesium level of 1.2 mg/dL, leading to early suspicion of thyroid storm. Further diagnostic evaluation revealed a positive test result for *Clostridium difficile * glutamate dehydrogenase antigen. An electrocardiogram (EKG) demonstrated atrial fibrillation with RVR and low voltage QRS complexes.

**Figure 1:**
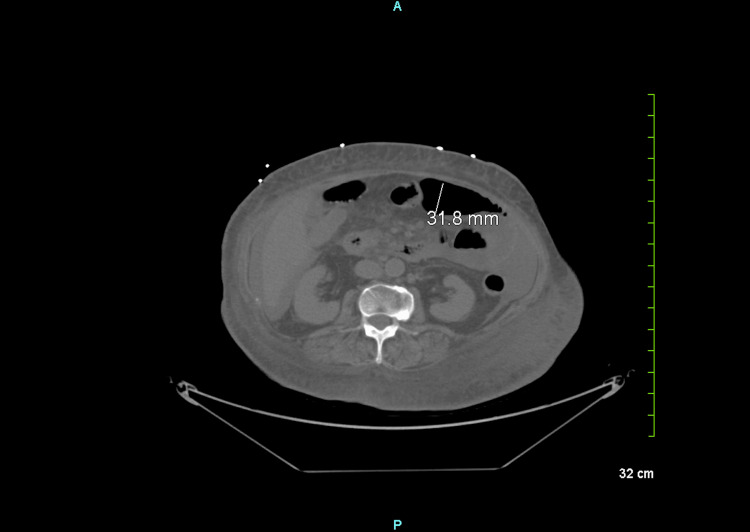
Abdominal CT scan without contrast depicts a small volume of free fluid diffusely distributed within the abdominal cavity, accompanied by mild body wall edema. Notably, the gallbladder is absent, while the liver, spleen, pancreas, adrenal glands, and kidneys exhibit unremarkable appearances. Few gas-distended loops of a small bowel are visualized without evidence of dilation, with a maximum small bowel diameter of 3.2 cm.

Further assessment via CT scan of the abdomen and pelvis revealed a slight dilation of the cardiac caliber and a moderate-sized pericardial effusion. CT scan also demonstrated the presence of diffuse free fluid, along with mild body wall edema and a limited number of gas-distended small bowel loops (Figure 1). Following the initial diagnosis of atrial fibrillation with RVR and suspected gastrointestinal infection, the patient failed to convert to sinus rhythm despite receiving Lopressor (metoprolol) thrice in the ED. A comprehensive thyroid workup was subsequently initiated, revealing markedly elevated levels of free T_3_ (18 pg/mL), free T_4_ (5.6 ng/dL), and undetectable levels of thyroid-stimulating hormone (TSH), consistent with thyroid storm. The patient's thyroid storm score was calculated to be 65, confirming the diagnosis.

Family members reported the patient's non-compliance with thyroid medication for several years. Immediate therapeutic intervention ensued, with the initiation of propranolol, methimazole, and methylprednisolone for thyroid storm management. Despite initial blood pressure reduction following Lopressor administration, the patient's deteriorating condition necessitated transfer to the ICU due to hemodynamic instability. Over the next few hours, her heart rate began to come under control but remained fluctuating.

**Figure 2:**
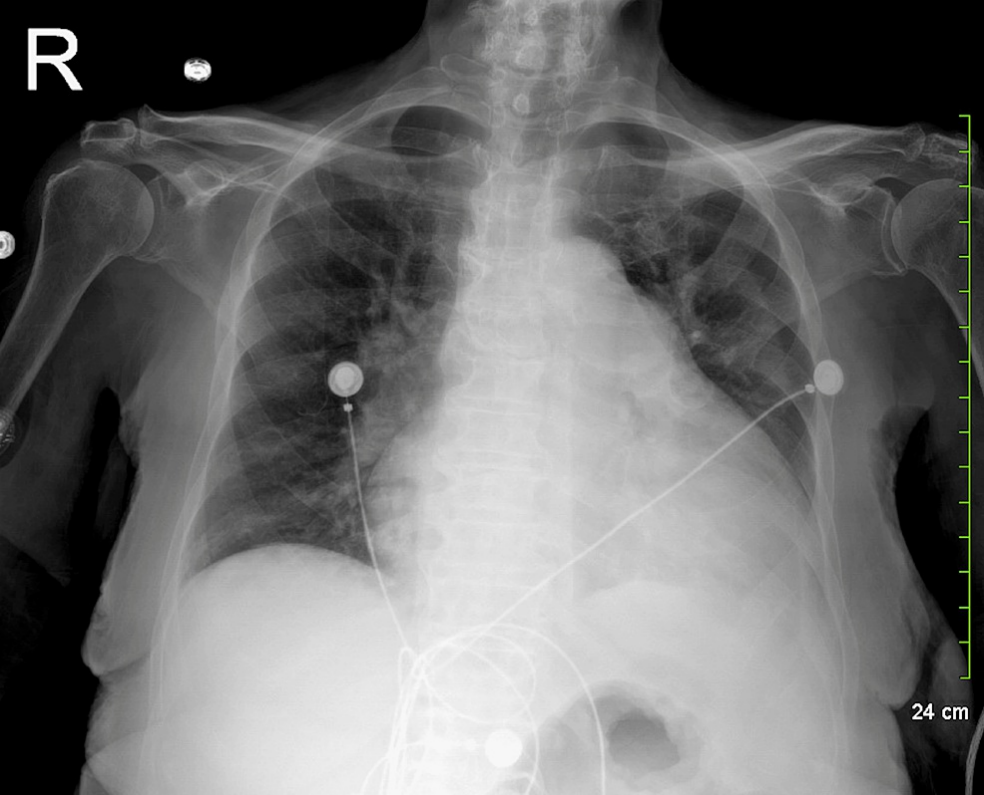
Chest X-ray (AP view) revealed an enlarged cardiac silhouette indicative of cardiomegaly and pericardial effusion, alongside pulmonary vascular congestion and mild left basilar opacities. No evidence of pneumothorax or significant pleural effusion, nor acute osseous abnormalities observed. AP: Antero-posterior

During her hospitalization, further investigations were conducted. Thyroglobulin levels were within normal limits, and the patient remained in rate-controlled atrial fibrillation. Additional imaging studies revealed cardiomegaly with mild pulmonary vascular congestion and basilar consolidation on chest X-ray (Figure 2). Left-heart cardiac catheterization demonstrated normal systolic function with mild coronary artery disease and an estimated ejection fraction greater than 60%. Transthoracic EKG findings included normal left ventricular chamber size with an ejection fraction of 40% to 45% and grade 1 diastolic dysfunction.

A nuclear medicine scan of the heart indicated a high probability of stress-induced myocardial infarction, suspected to be secondary to the prolonged uncontrolled thyrotoxicosis effect on the heart. Following the administration of anti-thyroid medications, normal sinus rhythm was restored from atrial fibrillation. With ongoing medical management, the patient's condition stabilized, and she was transitioned to regular inpatient floors with plans for close follow-up and optimization of thyroid function. By her eighth day of hospitalization, alkaline phosphatase (ALP), alanine transaminase (ALT), aspartate aminotransferase (AST), and total bilirubin levels had stabilized at 85, 18, 14, and 1.00 units/liter, respectively, throughout her treatment.

## Discussion

Thyroid storm, also known as thyroid crisis or thyrotoxic storm, is a rare but life-threatening endocrine emergency. It is an acute, severe manifestation of thyrotoxicosis that affects nearly all body systems [2]. This case highlights the challenges associated with diagnosing and managing thyroid storm in the clinical setting, particularly in patients with underlying Graves' disease.

Management of thyroid storm revolves around three main principles: reducing thyroid hormone production and release, blocking the peripheral effects of thyroid hormones, and supportive care to manage associated complications [9]. Beta-blockers, such as propranolol, serve as first-line agents to control adrenergic symptoms and reduce heart rate and blood pressure. Anti-thyroid medications, including methimazole and propylthiouracil, inhibit thyroid hormone synthesis and release. Additionally, glucocorticoids like methylprednisolone are often administered to attenuate the peripheral conversion of T_4_ to the more potent T_3_ and to modulate the inflammatory response associated with thyroid storm [10].

In our patient, the initial management was performed using propranolol, methimazole, and methylprednisolone, resulting in stabilization of her hemodynamic status. Despite these interventions, the patient's clinical course was complicated by persistent atrial fibrillation and the development of cardiovascular sequelae, including suspected stress-induced myocardial infarction. Atrial fibrillation is a common complication in thyroid disease and is often associated with underlying coronary artery disease. This combination can contribute to the progression of heart failure and worsen the overall prognosis [11]. The presence of coronary artery disease in patients with thyroid disease and atrial fibrillation necessitates careful evaluation and management to mitigate the risks and potential complications, including heart failure. This highlights the importance of continued monitoring and aggressive management in patients with thyroid storm, particularly in those with underlying cardiovascular comorbidities.

The relevance of this case lies in its illustration of the clinical challenges posed by thyroid storm, especially in the context of non-compliance with thyroid medication. The patient's prolonged untreated Graves' disease likely contributed to the development of thyroid storm, emphasizing the importance of patient education and adherence to treatment regimens in the management of thyroid disorders. The Burch-Wartofsky Point Scale (BWPS) is a scoring system that evaluates various clinical features, including fever, central nervous system disturbances, cardiovascular symptoms, gastrointestinal-hepatic dysfunction, and signs and symptoms of hyperthyroidism. Each feature is assigned a score based on its severity, and the total score is used to classify the condition as either thyrotoxicosis or thyroid storm. A score of 25 or higher indicates probable thyroid storm, while a score of 45 or higher strongly suggests the diagnosis [12]. By providing an objective measure of severity, the BWPS aids clinicians in making timely and accurate decisions regarding the diagnosis and management of patients with thyrotoxicosis. Moreover, this case emphasizes the need for a multidisciplinary approach, involving endocrinologists, cardiologists, and critical care specialists, to optimize patient outcomes and prevent complications associated with thyroid storm [13].

## Conclusions

Untreated thyrotoxicosis can lead to thyroid storm, a severe and potentially fatal condition. This case highlights the importance of considering thyroid storm in the differential diagnosis of patients presenting with abdominal symptoms, atrial fibrillation, and new-onset high-output cardiac failure, especially in those with a history of untreated thyroid disease. Early recognition and aggressive management using the five Bs of thyroid storm treatment-block synthesis with anti-thyroid drugs, block release with iodine, block T_4_ to T_3_ conversion with high-dose propylthiouracil, propranolol, or corticosteroids, beta blockers, and block enterohepatic circulation with cholestyramine-are crucial for improving outcomes and preventing morbidity and mortality. Close follow-up and optimization of thyroid function are essential in the long-term management of these patients.

## References

[1] Chiha M, Samarasinghe S, Kabaker AS (2015). Thyroid storm: an updated review. J Intensive Care Med.

[2] Pandey R, Kumar S, Kotwal N (2020). Thyroid storm: Clinical manifestation, pathophysiology, and treatment. Goiter-Causes Treatment.

[3] Soares MN, Borges-Canha M, Neves C, Neves JS, Carvalho D (2023). The role of Graves' disease in the development of thyroid nodules and thyroid cancer. Eur Thyroid J.

[4] Antonelli A, Ferrari SM, Ragusa F (2020). Graves' disease: Epidemiology, genetic and environmental risk factors and viruses. Best Pract Res Clin Endocrinol Metab.

[5] Inman BL, Long B (2023). Thyrotoxicosis. Emerg Med Clin North Am.

[6] Ritter K, Wolfe C (2020). Thyroid storm. J Educ Teach Emerg Med.

[7] Bacuzzi A, Dionigi G, Guzzetti L, De Martino AI, Severgnini P, Cuffari S (2017). Predictive features associated with thyrotoxic storm and management. Gland Surg.

[8] De Leo S, Lee SY, Braverman LE (2016). Hyperthyroidism. Lancet.

[9] Wiersinga WM, Poppe KG, Effraimidis G (2023). Hyperthyroidism: aetiology, pathogenesis, diagnosis, management, complications, and prognosis. Lancet Diabetes Endocrinol.

[10] Himes CP, Ganesh R, Wight EC, Simha V, Liebow M (2020). Perioperative evaluation and management of endocrine disorders. Mayo Clin Proc.

[11] Batta A, Hatwal J, Batta A, Verma S, Sharma YP (2023). Atrial fibrillation and coronary artery disease: an integrative review focusing on therapeutic implications of this relationship. World J Cardiol.

[12] De Almeida R, McCalmon S, Cabandugama PK (2022). Clinical review and update on the management of thyroid storm. Mo Med.

[13] Wijeratne S, Chong C, Kirthinanda DS (2022). Anaesthesiology perspective on a multidisciplinary approach to optimal perioperative management of a patient with giant peptic ulcer perforation caused by the physiological stress of a thyroid storm necessitating emergent laparotomy. BMJ Case Rep.

